# Characteristics of the gut microbiota and serum metabolites in postmenopausal women with reduced bone mineral density

**DOI:** 10.3389/fcimb.2024.1367325

**Published:** 2024-06-07

**Authors:** Litao Yan, Xianfeng Wang, Tiantian Yu, Zhiming Qi, Huan Li, Hao Nan, Kun Wang, Di Luo, Fei Hua, Wendong Wang

**Affiliations:** ^1^ Department of Articular Orthopaedics, The First People’s Hospital of Changzhou, The Third Affiliated Hospital of Soochow University, Changzhou, China; ^2^ Department of Orthopedic Surgery, Beijing Jishuitan Hospital Guizhou Hospital, Guiyang, China; ^3^ Department of Gynaecology and Obstetrics, Dalian Municipal Woman and Children’s Medical Center, Dalian, China; ^4^ Department of Articular Orthopaedics, The Second People’s Hospital of Dalian, Dalian, China; ^5^ Changzhou Medical Center, Nanjing Medical University, Nanjing, China; ^6^ Department of Clinical Laboratory, The Second People’s Hospital of Dalian, Dalian, China; ^7^ Department of Endocrinology and Metabolism, The First People’s Hospital of Changzhou, The Third Affiliated Hospital of Soochow University, Changzhou, China

**Keywords:** osteoporosis, gut microbiota, serum metabolites, postmenopausal women, tryptophan-indole metabolism

## Abstract

**Introduction:**

Emerging evidence suggests that the gut microbiota is closely associated with bone homeostasis. However, little is known about the relationships among the bone mineral density (BMD) index, bone turnover markers, and the gut microbiota and its metabolites in postmenopausal women.

**Methods:**

In this study, to understand gut microbiota signatures and serum metabolite changes in postmenopausal women with reduced BMD, postmenopausal individuals with normal or reduced BMD were recruited and divided into normal and OS groups. Feces and serum samples were collected for 16S rRNA gene sequencing, liquid chromatography coupled with mass spectrometry (LC-MS)-based metabolomics and integrated analysis.

**Results:**

The results demonstrated that bacterial richness and diversity were greater in the OS group than in the normal group. Additionally, distinguishing bacteria were found among the two groups and were closely associated with the BMD index and bone turnover markers. Metabolomic analysis revealed that the expression of serum metabolites, such as etiocholanolone, testosterone sulfate, and indole-3-pyruvic acid, and the corresponding signaling pathways, especially those involved in tryptophan metabolism, fatty acid degradation and steroid hormone biosynthesis, also changed significantly. Correlation analysis revealed positive associations between normal group-enriched *Bacteroides* abundance and normal group-enriched etiocholanolone and testosterone sulfate abundances; in particular, *Bacteroides* correlated positively with BMD. Importantly, the tryptophan-indole metabolism pathway was uniquely metabolized by the gut bacteria-derived *tnaA* gene, the predicted abundance of which was significantly greater in the normal group than in the control group, and the abundance of *Bacteroides* was strongly correlated with the *tnaA* gene.

**Discussion:**

Our results indicated a clear difference in the gut microbiota and serum metabolites of postmenopausal women. Specifically altered bacteria and derived metabolites were closely associated with the BMD index and bone turnover markers, indicating the potential of the gut microbiota and serum metabolites as modifiable factors and therapeutic targets for preventing osteoporosis.

## Introduction

As a metabolic bone disease, osteoporosis is characterized by decreased bone mineral density (BMD) and microarchitectural deterioration (Gopinath, 2023). In elderly adults with impaired bone metabolism, fractures are more likely to occur, causing pain, disability, and loss of function (Liang et al., 2022). It is estimated that 10% of the global population suffers from osteoporosis, with 30% of those suffering from it being postmenopausal women older than 50 years of age (Black and Rosen, 2016; Yu and Xia, 2019). However, the current understanding of the pathogenesis of osteoporosis is insufficient for developing drugs that can completely prevent the occurrence and development of osteoporosis (Reid and Billington, 2022). As a result, identifying effective preventive intervention strategies to reduce the risk of fracture and diagnosing osteopenia early are important clinical issues (Song et al., 2022).

The gut microbiome inhabits the gastrointestinal tract and consists of approximately 10 trillion bacteria. The gut microbiota can interact with various organs and systems and is an important medium between the host and microorganisms (Brown et al., 2023). New high-throughput sequencing technologies have made it possible to analyze metabolic characteristics of intestinal microbial communities on a large scale, providing new directions for treating various metabolic disorders. There is growing evidence that the gut microbiota or its metabolites have an impact on bone health by regulating immune, vascular, endocrine, intestinal, and nervous systems (Ding et al., 2020; Song et al., 2022; Xu et al., 2022).. Postmenopausal women exhibit altered gut microbiomes, as well as osteopenia and osteoporosis (Rettedal et al., 2021). The gut microbiota-bone axis suggests that *Prevotella histicola* can specifically protect against estrogen deficiency-induced bone loss (Wang et al., 2021). Bioavailable isoflavone and probiotic treatment improve bone status and estrogen metabolism in postmenopausal osteopenic women, according to a randomized controlled trial (Lambert et al., 2017). It follows that gut microbes play a direct role in postmenopausal osteoporosis bone metabolism regulation. However, the specific mechanisms involved in the interaction between the gut microbiota, serum metabolites, and bone metabolism remain unknown.

This project used an integrated approach combining 16S rRNA gene sequencing with LC-MS-based metabolomics analysis of blood and feces to determine whether specific gut microbiota and their metabolites are associated with reduced BMD in postmenopausal women. In this study, we evaluated the species and abundance of the gut microbiota and their interactions with metabolites in postmenopausal women with reduced BMD, thus providing new insights into the pathogenesis and therapeutic targets of postmenopausal osteoporosis.

## Materials and methods

### Study subjects

This study was approved by the Ethics Committee of The Second People’s Hospital of Dalian (No.2022.174X). For gut microbiota analysis, postmenopausal subjects with normal BMD (normal group, n=38) or reduced BMD (OS group, n=25) were recruited. Clinical information was presented in **Table 1**. For metabolomics analysis, normal group (n=61) and OS group (n=61) were recruited. All subjects participating in the study were over 50 years of age and had undergone menopause. Detailed clinical information is presented in **Table 2**. The study did not include participants with cancer, kidney disease, metabolic or genetic bone disease, digestive system disease, psychiatric illness, or those who had taken antibiotics within 3 months or those taking medications that might affect bone metabolism.

**Table 1:** Clinical information of the participants for gut microbiota analysis.

Characteristics	Normal (n=38)	OS (n=25)	P-value
BMI (kg/m^2^)	25.17 ± 3.51	23.35 ± 2.82	NS
Age (years)	65.21 ± 7.55	63.76 ± 6.63	NS
LS BMD (g/cm^2^)	1.24 ± 0.18	0.99 ± 0.11	<0.001
LS T-score	1.24 ± 1.54	-1.02 ± 0.94	<0.001
LS Z-score	2.02 ± 1.57	0.46 ± 1.01	<0.001
Neck BMD (mg/cm^2^)	955.16 ± 172.41	778.04 ± 165.85	<0.001
Neck T-score	0.67 ± 1.45	-0.72 ± 1.37	<0.001
Neck Z-score	1.67 ± 1.70	0.56 ± 1.38	<0.01
Hip BMD (mg/cm^2^)	1015.63 ± 116.46	806.44 ± 156.85	<0.001
Hip T-score	0.65 ± 0.92	-0.94 ± 1.21	<0.001
Hip Z-score	1.16 ± 1.14	-0.08 ± 1.67	<0.001
ALP (U/L)	74.10 ± 21.13	93.43 ± 25.91	<0.01
VD (ng/mL)	27.74 ± 9.58	21.78 ± 6.18	<0.01
PTH (pg/mL)	33.20 ± 11.67	32.28 ± 10.73	NS
T-PINP (ng/mL)	46.22 ± 16.85	71.52 ± 29.51	<0.001
β-CTX (pg/mL)	504.07 ± 192.87	739.78 ± 263.93	<0.001
N-MID (ng/mL)	14.98 ± 5.21	23.38 ± 10.74	<0.001
E2(pmol/L)	52.11 ± 16.59	42.89 ± 10.37	<0.05

Values are mean ± SD for continuous variables and the P value is based on the Wilcoxon rank-sum test.

NS, nonsignificance.

**Table 2:** Clinical information of the participants for serum metabolites analysis.

Characteristics	Normal (n=61)	OS (n=61)	P-value
BMI (kg/m^2^)	25.52 ± 3.42	23.82 ± 3.15	NS
Age (years)	64.25 ± 7.25	67.41 ± 7.94	NS
LS BMD (g/cm^2^)	1.24 ± 0.15	0.96 ± 0.12	<0.001
LS T-score	1.18 ± 1.33	-1.27 ± 0.98	<0.001
LS Z-score	2.07 ± 1.42	0.51 ± 1.04	<0.001
Neck BMD (mg/cm^2^)	949.30 ± 159.88	714.18 ± 143.89	<0.001
Neck T-score	0.67 ± 1.34	-1.25 ± 1.21	<0.001
Neck Z-score	1.68 ± 1.57	0.23 ± 1.21	<0.001
Hip BMD (mg/cm^2^)	1007.59 ± 120.66	755.21 ± 135.88	<0.001
Hip T-score	0.60 ± 0.95	-1.33 ± 1.05	<0.001
Hip Z-score	1.17 ± 1.08	-0.24 ± 1.01	<0.001
ALP (U/L)	73.51 ± 23.86	91.32 ± 23.68	<0.001
VD (ng/mL)	20.17 ± 10.68	20.23 ± 8.30	<0.01
PTH (pg/mL)	31.93 ± 12.45	34.72 ± 12.12	NS
T-PINP (ng/mL)	49.98 ± 27.34	70.75 ± 30.21	<0.001
β-CTX (pg/mL)	507.54 ± 193.38	725.53 ± 300.86	<0.001
N-MID (ng/mL)	15.62 ± 5.07	22.28 ± 9.51	<0.001
E2(pmol/L)	56.08 ± 19.40	45.61 ± 15.09	<0.01

Values are mean ± SD for continuous variables and the P value is based on the Wilcoxon rank-sum test.

NS, nonsignificance.

### Clinical data and sample collection

All subjects were interviewed and measured for age, height, and weight, as well as their body mass index (BMI). Fresh feces and blood samples were collected after more than six hours of fasting in the morning.

After finishing serological testing, all collected serum and feces samples were transported to -80°C freezer for further analysis. Serum levels of total 25 (OH) vitamin D (VD), alkaline phosphatase (ALP), osteocalcin (N-MID), C-terminal peptide of type I collagen (β-CTX), N-terminal propeptide of type 1 procollagen (T-P1NP), and parathyroid hormone (PTH) were measured. BMD, T-score and Z-score in the lumbar spine (L1-4, LS), femoral neck (Neck) and total hip joint (femoral neck, trochanteric and intertrochanteric areas, Hip) were measured using a dual-energy X-ray absorptiometry scanner.

### Microbiota 16S rRNA gene sequencing

Total genome DNA from feces samples was extracted using CTAB/SDS method. DNA concentration and purity was monitored on 1% agarose gels. According to the concentration, DNA was diluted to l ug/μL using sterile water. Mix same volume of IX loading buffer (contained SYB green) with PCR products and operate electrophoresis on 2% agarose gel for detection. PCR products was mixed in equidensity ratios. Then, mixture PCR products was purified with Qiagen Gel Extraction Kit (Qiagen, Germany). Fecal DNA was isolated and the V4 hyper variable regions of the bacterial 16S rRNA gene was amplified by primers 515F (GTGCCAGCMGCCGCGGTAA) and 806R (GGACTACHVGGGTWTCTAAT). After denaturation for 1 minute at 98°C, 30 cycles were performed, each lasting 10 seconds, followed by annealing at 50°C for 30 seconds and elongation at 72°C for 30 seconds. As soon as PCR products were purified, TruSeq DNA PCR-free sample preparation kits (Illumina, USA) and index codes were added to create sequencing libraries. At last, the libraries were sequenced on an Illumina NovaSeq platform and 250 bp paired-end reads were generated. The QIIME V.2.0 pipeline was used after barcodes were taken off the sequences (Hall and Beiko, 2018). Random selection of reads was used to reduce ASVs of each sample to 10000 (Valles-Colomer et al., 2022). ASV taxonomy was based on the Silva 138 SSURef NR99 16S rRNA gene reference database (Quast et al., 2013; Zhao et al., 2022).

### Analysis of gut microbiota profile

Alpha diversity of gut microbiota was estimated by R package Vegan. And the beta diversity was estimated via vegdist function of Vegan R package based on the genus levels’ Bray-Curtis distance matrix (Lloyd-Price et al., 2019). The significant difference of the beta diversity was statistic by PERMANOVA test (adonis function). And the bacterial composition was analyzed by phyloseq R package (McMurdie and Holmes, 2013). Further, the LEfSe (Linear Discriminant Analysis Effect Size) analyze was performed via microbiomeMarker R package (Cao et al., 2022). Moreover, the predicted function of microbiota was an estimated by PICRUSt2 (Liu et al., 2016; Douglas et al., 2020).

### Analysis of serum metabolomics

A difference in serum metabolite signatures was detected between normal and OS groups by LC-MS. For the identification of metabolites, processed data, such as m/z, RT, and normalized peak areas, were imported into SIMCA. Metabolites were identified using the HMDB (Human Metabolome Database). The HMDB database was adopted to map and identify the metabolites. By using R package ropls to identify the metabolites changes within groups, partial least squares discriminant analysis (PLS-DA) was used to analyze the abundance of significant metabolites with projection (VIP) > 1 and p value (Wilcoxon test) < 0.05. The enrichment pathway between normal and OS serum metabolites profiles was analyzed by MetaboAnalyst 5.0 (Pang et al., 2021).

### Correlation analysis

Using Spearman’s correlation analysis, more than 20 variables including clinical variables and differences in microbiome were analyzed for the correlation between predicted function and changes in bacteria, microbiota, and metabolites. R package “psych”, version 2.4.1 was used in the correlation analysis.

### Marker panel for OS individuals

In the discovery dataset, linear regression model was built based on the signatures of microbiota or metabolites of OS subjects (Liang et al., 2020). And the possibility of model was estimated by predict function in both discovery and validation sets. Besides, the accuracy of marker panel for discriminate the OS participants from normal cases in both discovery and validation sets by R package pROC (Robin et al., 2011).

### Co-occurrence network analysis

The co-occurrence network of gut microbiota in normal and OS groups was established by R package ggClusterNet based on top 500 ASVs (Wen et al., 2022). And the network vulnerability was estimated via ggClusterNet R package. Besides, the average variation degree (AVD) of the network was analyzed as previous described (Xun et al., 2021).

### Statistical analyses

All statistical analyses and graphical representations of the study were performed via R. Data were expressed in mean ± SEM unless otherwise stated. We utilized Wilcox and chi-squared test to evaluate the difference between two groups for continuous and categorical variables, respectively. Significant difference between two groups at a confidence level of 0.05.

## Results

### Characteristics of the gut microbiota profile and metabolic function of OS patients

**Figure 1:**
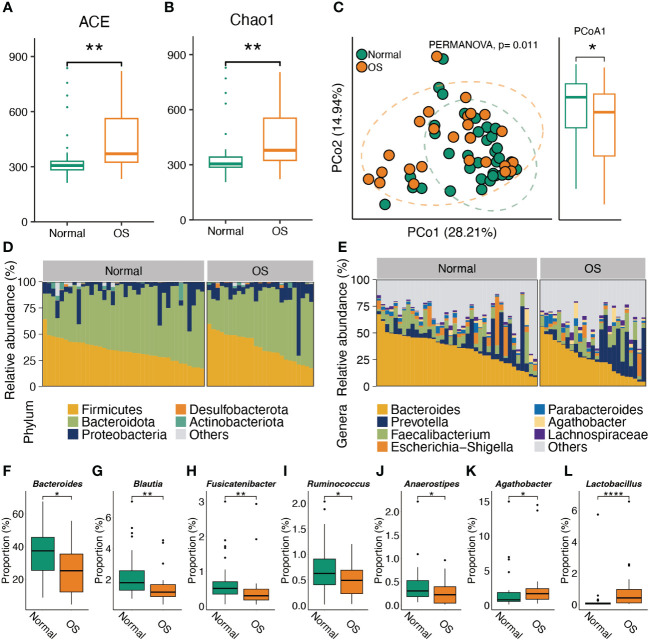
Characteristic the gut microbiota composition. **(A, B)** The alpha diversity comparison of microbiota between two groups. **(C)** PCoA analysis of gut microbes at genus level. **(D, E)** The gut microbiota composition in normal and OS cases at phylum and genus levels, respectively. **(F–L)** The significant difference of gut bacteria at genus level. *: p<0.05, **: p<0.01.

As shown in **Figures 1A, B**, the gut bacterial ACE and Chao1 indices of OS participants were greater than those of the healthy individuals. In addition, PCoA of the genus abundance of the gut microbiota revealed clear differences between the two groups (PERMANOVA test, *P*<0.05) (**Figure 1C**). The difference arose from PCoA1 (*P*<0.05). At the phylum level, there was no significant difference between the two groups (**Figure 1D**). At the genus level, the proportions of *Bacteroides*, *Blautia*, *Fusicatenibacter*, *Ruminococcus*, and *Anaerostipes* in the OS group were lower than those in the normal group (**Figures 1E–J**). However, higher proportions of the *Agathobacter* and *Lactobacillus* genera were observed in OS patients (**Figures 1K, L**).

### Identification of the signatures of the gut microbiota profile and metabolic function in OS patients

**Figure 2:**
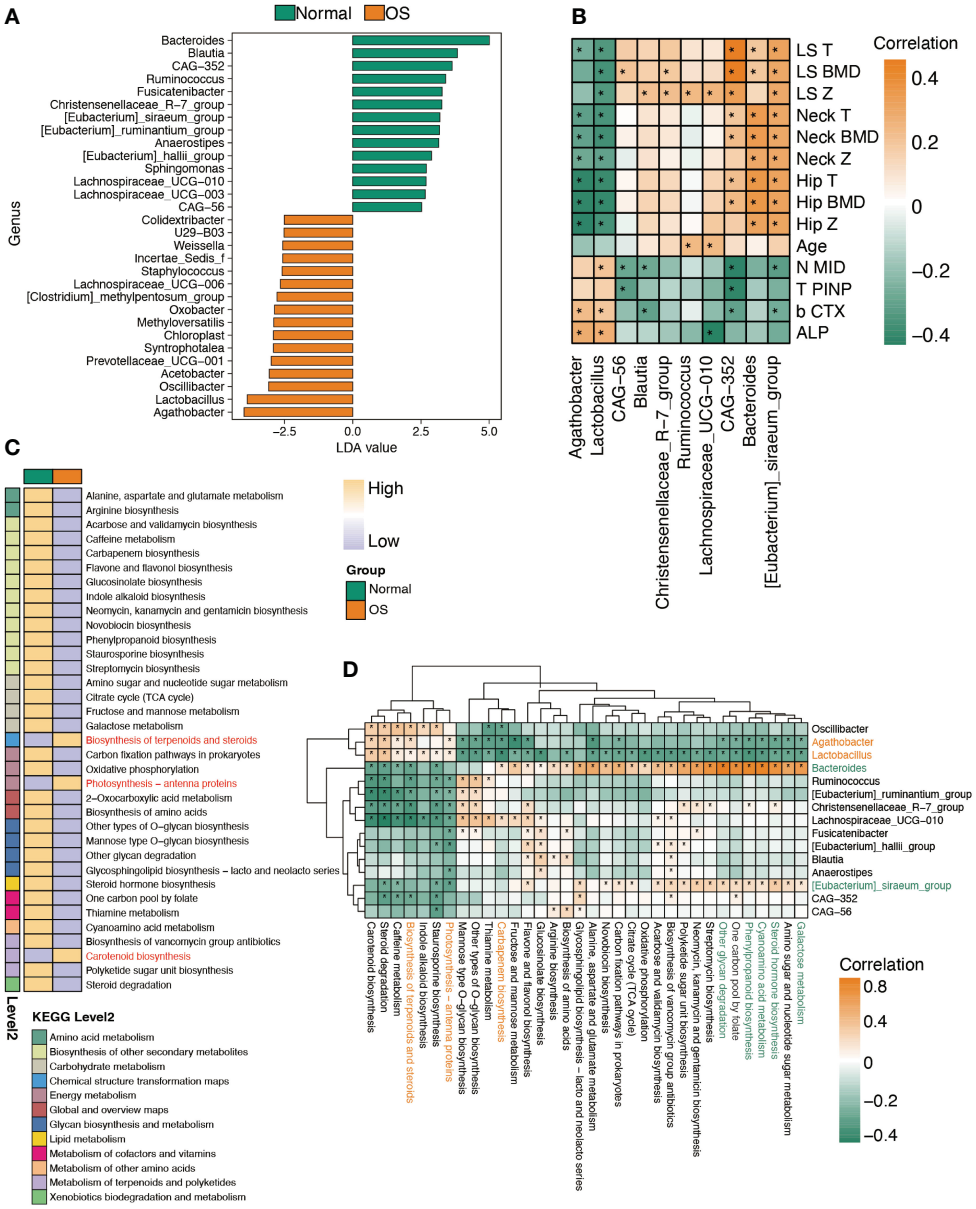
Characteristic the function of gut microbiota. **(A)** LDA analysis of gut microbiota between two groups at genus level. **(B)** Spearman’s correlation between significantly altered bacteria and clinical index. **(C)** The remarkably difference predicted function of bacteria between two groups. **(D)** The correlation analysis of predicted function and bacteria abundance. *: p<0.05.

To identify the key gut bacteria for distinguishing the two groups, LEfSe was performed to analyze the taxa. As shown in **Figure 2A**, the OS individuals were characterized by enrichment of the genera *Agathobacter*, *Lactobacillus*, *Oscillibacter*, and *Prevotellaceae_UGG-001*. The *Agathobacter* and *Lactobacillus* genera were negatively correlated with the bone density index (**Figure 2B**). Notably, the *Agathobacter* and *Lactobacillus* genera strongly correlated with the bone loss markers β-CTX and ALP (**Figure 2B**). However, enrichment of the genera *Bacteroides*, *CAG-352*, and *[Eubacterium]_siraeum_group* in normal individuals was positively correlated with BMD (**Figure 2B**). Furthermore, the predicted metabolic function analysis of the gut microbiota showed that the biosynthesis of terpenoids and steroids, photosynthesis−antenna proteins and carotenoid biosynthesis pathways were enhanced in OS patients (**Figure 2C**). Importantly, the metabolic functions of terpenoid and steroid biosynthesis and photosynthesis-antenna proteins were positively correlated with the *Agathobacter* and *Lactobacillus* genera, which dominate in OS individuals (**Figure 2D**). The genera *Bacteroides* and *[Eubacterium]_siraeum_group* were negatively associated with the abovementioned pathways (**Figure 2D**). However, the genera *Bacteroides* and *[Eubacterium]_siraeum_group* were strongly correlated with galactose metabolism, steroid hormone biosynthesis, cyanoamino acid metabolism, phenylpropanoid biosynthesis, and one carbon pool generated by folate pathways.

### Characteristic gut bacterial associations and community stability of OS individuals

**Figure 3:**
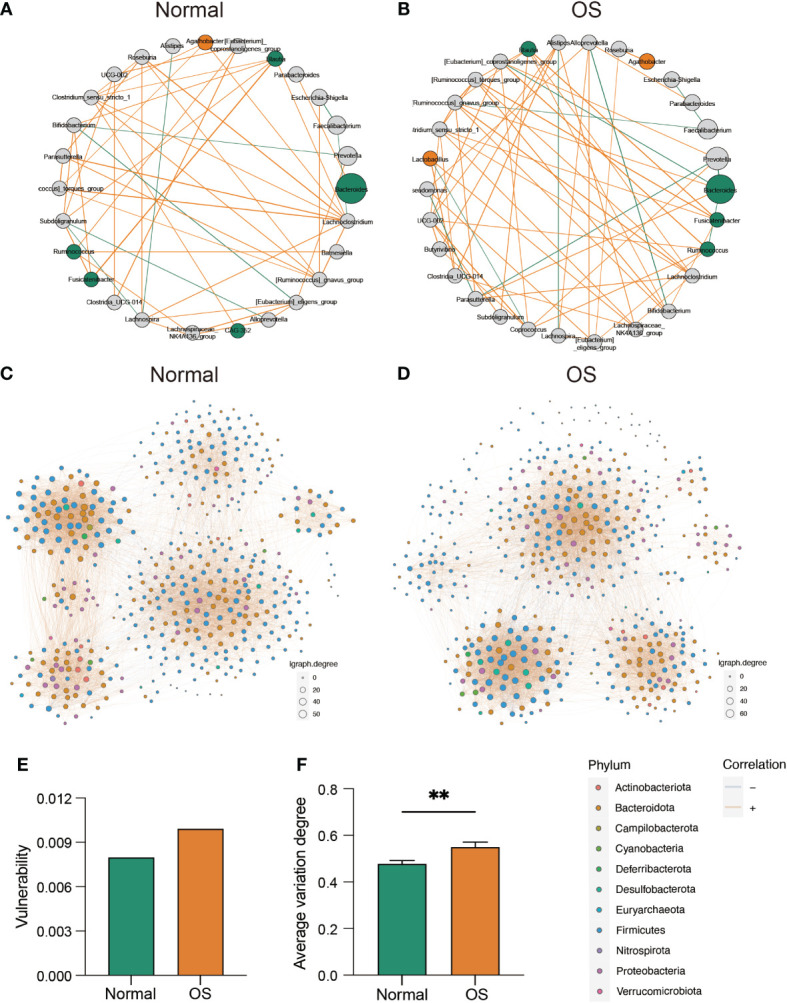
The evaluation of gut microbiota interaction and community stability. **(A, B)** The correlation ship among normal and OS cases, respectively. The orange and green dote indicated bacterial which dominated in OS and normal cases, respectively. **(C, D)** The bacterial co-occurrence network in two groups at ASVs level. **(E)** The vulnerability of co-occurrence network between two groups. **(F)** The comparison of average variation degree between two groups. **: p<0.01.

We next analyzed the associations of gut bacteria. As shown in **Figures 3A, B**, a clear difference in the correlation network at the genus level was observed between the two groups. Specifically, *Bacteroides* was correlated with *Parabacteroides* only in the normal group (**Figure 3A**). However, in the OS group, the genus *[Eubacterium]_coprostanoligenes_group* was negatively correlated with *Bacteroides* (**Figure 3B**). In addition, the enrichment of *Lactobacillus* and *Agathobacter* in OS patients was positively correlated with that of *Clostridia_UCG-014*, *Lachnospiraceae_NK4A136_group*, and *Roseburia* (**Figure 3B**). Furthermore, we analyzed the stability of the gut microbiota community at the ASV level (**Figures 3C, D**). Compared to that in the normal group, the vulnerability of the microbiota network in the OS group was increased (**Figure 3E**). Consistently, the average variation degree was also significantly greater in the OS group (**Figure 3F**).

### The serum metabolome revealed a distinct metabolism in OS patients

**Figure 4:**
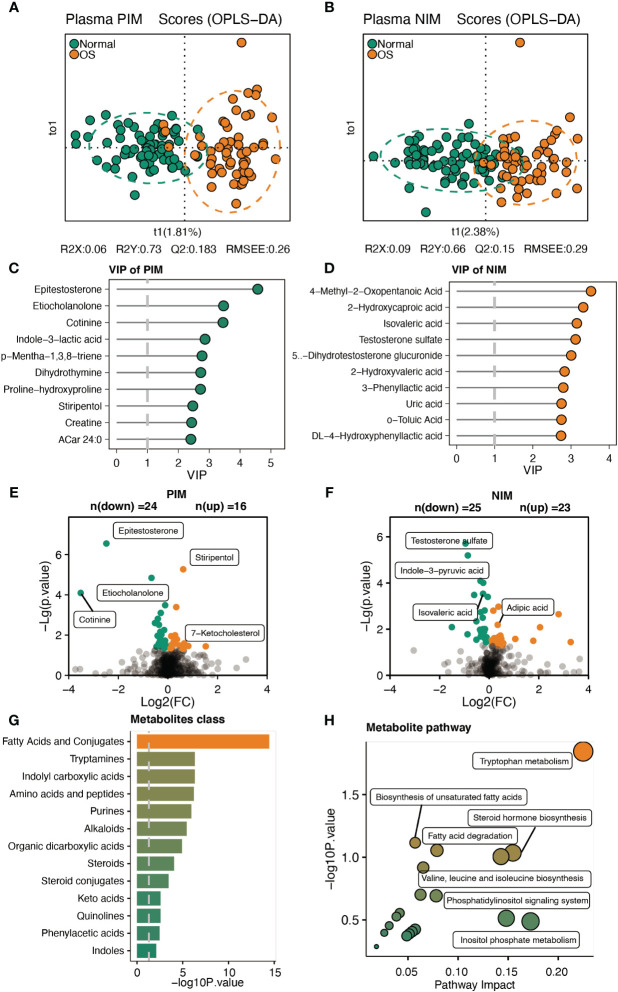
Characteristic of the serum metabolites. **(A, B)** The OPLS-DA analysis of serum metabolites between two groups in PIM and NIM, respectively. **(C, D)** The VIP score of metabolites in PIM and NIM, respectively. **(E, F)** Volcano plots indicated the different metabolites in PIM and NIM, respectively. **(G, H)** The metabolites’ sets and KEGG pathway enrichment based on differ metabolites.

We further analyzed the serum metabolites by analyzing the untargeted metabolome. As shown in **Figures 4A, B**, the NIM and PIM showed distinct clustering patterns compared to samples from individuals with normal survival. The VIP scores for the metabolites revealed that epitestosterone and 4−methyl−2−oxopentanoic acid contributed significantly to the separation of the PIM and NIM groups, respectively (**Figures 4C, D**). In addition, a total of 49 (PIM: 24, NIM: 25) metabolites were downregulated, and 39 (PIM: 16, NIM: 23) metabolites were upregulated in the OS group (**Figure 4E, F**). Specifically, the levels of epitestosterone, etiocholanolone, testosterone sulfate, indole−3-pyruvic acid and isovaleric acid were significantly decreased in OS patients (**Figures 4E, F**). However, the levels of stiripentol, 7-ketocholesterol and adipic acid were markedly increased in OS patients (**Figures 4E, F**). Analyses of metabolic sets revealed that altered metabolites contained a considerable amount of fatty acids, conjugates, tryptamines, indolyl carboxylic acids, amino acids, and peptides (**Figure 4G**). Differentially abundant metabolites were enriched in pathways such as tryptophan metabolism, unsaturated fatty acid biosynthesis, steroid hormone biosynthesis, fatty acid degradation, and valine, leucine, and isoleucine biosynthesis in OS patients (**Figure 4H**).

### Integrated analysis of the host- and gut bacteria-derived serum metabolites.

**Figure 5:**
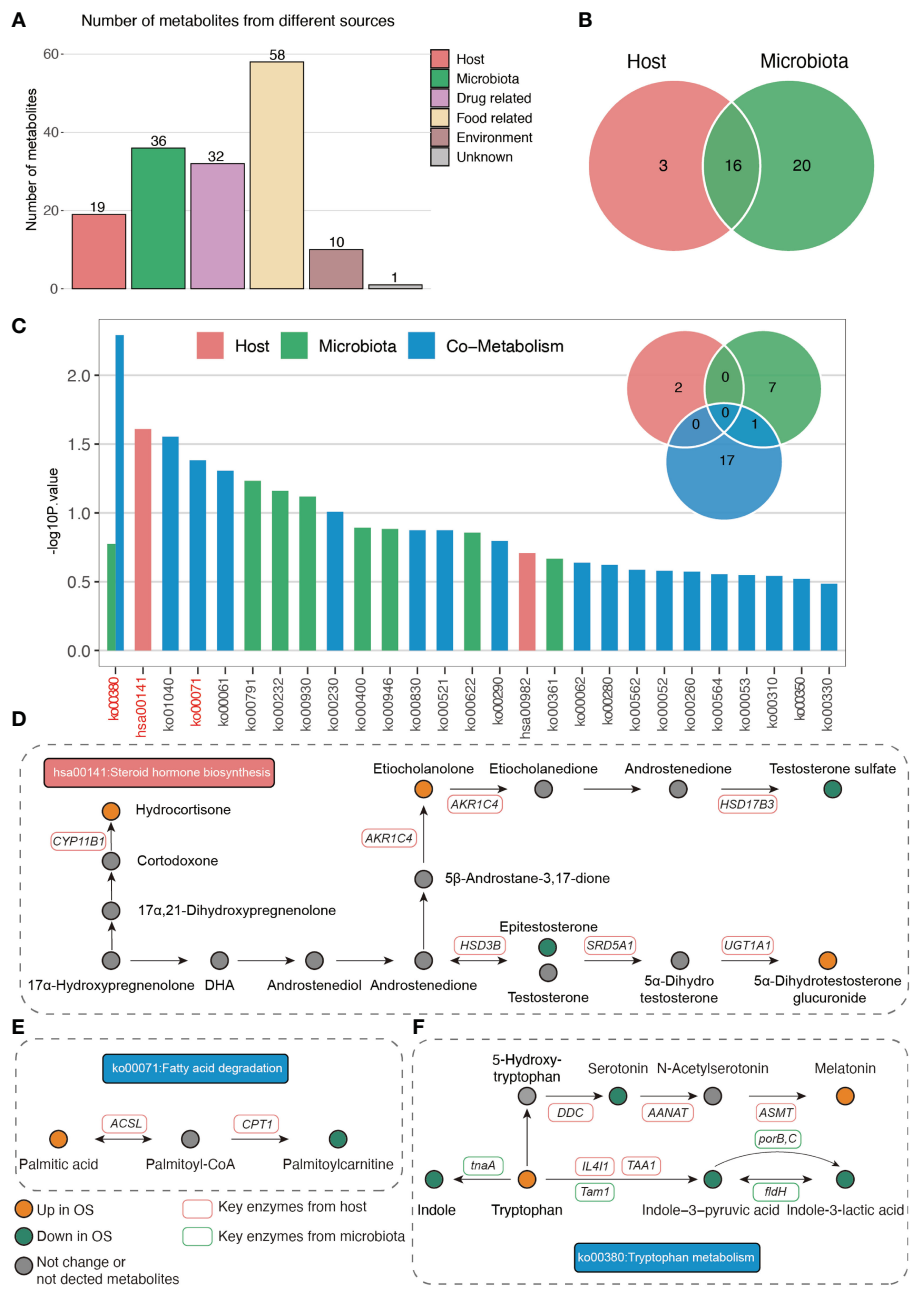
The origin analysis of serum metabolites. **(A)** The source of differ serum metabolites between two groups. **(B)** Venn plot indicated the number of metabolites origin from host and gut microbiota. **(C)** The pathway enrichment based on host and gut microbiota derived metabolites. **(D–F)** The illustration of significantly enriched pathways.

Next, we analyzed the origins of the serum metabolites that were significantly different between the two groups. As shown in **Figure 5A**, 19 and 36 plasma metabolites were identified as originating from the host and gut microbiomes, respectively. Sixteen metabolites were derived from the host and gut bacteria (**Figure 5B**). Moreover, pathway enrichment analysis of the metabolites indicated that steroid hormones were uniquely metabolized by the host (**Figures 5C, D**). In addition, fatty acid degradation occurs via cometabolism by the host and gut microbiota (**Figures 5C, E**). Importantly, host- and gut microbiota-derived metabolites jointly participate in the Trp metabolism pathway (**Figures 5C, F**). The plasma-derived indole was uniquely metabolized by the gut bacteria-derived *tnaA* gene (**Figure 5F**). In addition, the gut microbiota *Tam1* and host *IL4I1* and *TAA1* commonly facilitate the production of indole-3-pyruvic acid from tryptophan (**Figure 5F**). In addition, the gut bacteria *fldH*, *porB* and *porC* promote the conversion of indole-3-pyruvic acid to indole-3-lactic acid (**Figure 5F**).

### Association analysis of the gut microbiota, serum metabolites and BMD indices

**Figure 6:**
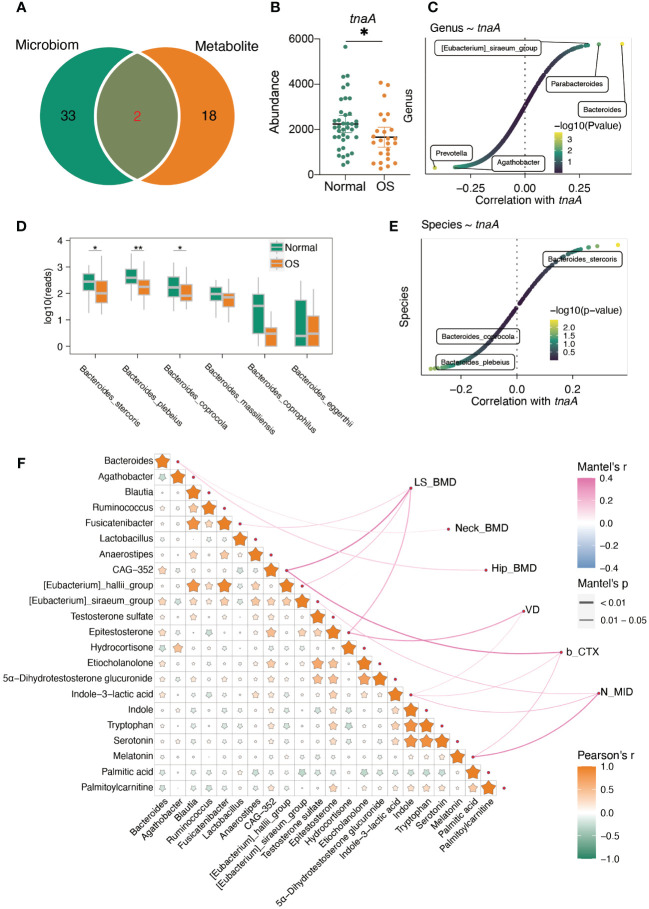
The interaction of gut microbiota and serum metabolites. **(A)** The altered predicted function of gut microbiota and significantly enriched pathway based on metabolites. **(B)** The predicted gut microbiota *tnaA* gene abundance based on PICRUTS2. **(C)** The correlation between *tnaA* gene abundance and gut bacteria. **(D)** The comparison of *Bacteroides* at species level. **(E)** The correlation between *tnaA* gene abundance and *Bacteroides* species. **(F)** Mantel’s analysis the interaction of differ gut microbiota, serum metabolites and clinical index. *: p<0.05, **: p<0.01.

Moreover, the integrated analysis of pathways enriched in genes related to altered gut bacteria and serum metabolites showed that only the galactose metabolism and steroid hormone biosynthesis pathways overlapped between the microbiome and metabolites (**Figure 6A**). In addition, the predicted abundance of the *tnaA* gene in the gut microbiota of OS patients was significantly lower than that in that of normal individuals (**Figure 6B**). The *Bacteroides* genus was strongly correlated with the *tnaA* gene (**Figure 6C**). We further compared the abundance of gut microbes that belong to the *Bacteroides* genus at the species level. As shown in **Figure 6D**, the abundance of *Bacteroides stercoris*, *Bacteroides plebeius*, and *Bacteroides coprocola* significantly decreased in the OS group. However, only *Bacteroides stercoris was* positively correlated with the *tnaA* gene (**Figure 6E**).

In addition, the integrated correlation analysis showed that *Bacteroides* was positively correlated with epitestosterone, etiocholanolone, testosterone sulfate, 5a-dihydrotestosterone glucuronide and indole-3-lactic acid (**Figure 6F**). However, hydrocortisone, palmitic acid and palmitoylcarnitine were negatively correlated with *Bacteroides* genera (**Figure 6F**). Importantly, the *Bacteroides* genus was positively correlated with neck BMD and hip BMD (**Figure 6F**). In addition, epitestosterone, etiocholanolone, 5α-dihydrotestosterone glucuronide, indole-3-lactic acid, indole, tryptophan, and serotonin were positively correlated with palmitoylcarnitine but negatively correlated with palmitic acid (**Figure 6F**). The level of epitestosterone strongly correlated with the levels of etiocholanolone, 5α-dihydrotestosterone glucuronide, indole-3-lactic acid, indole, tryptophan, and serotonin (**Figure 6F**). The serum epitestosterone concentration was positively correlated with VD and LS BMD (**Figure 6F**).

### Combined biomarkers for discriminating OS patients from normal participants

**Figure 7:**
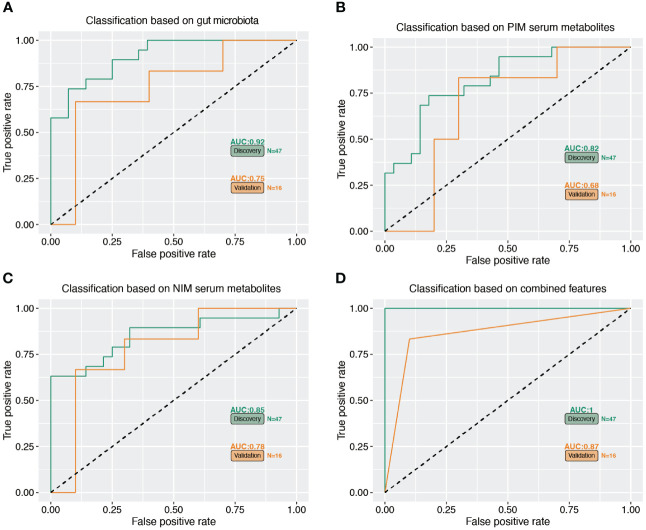
Multiple markers for diagnosis of reduced BMD. **(A–C)** The separated gut microbiota, serum metabolites of PIM and NIM diagnose OS from normal subjects. **(D)** The accuracy of combined marker panels for diagnose OS from normal subjects.

We further constructed a linear regression model based on the OS signature-related gut microbiota (*Bacteroides*, *Agathobacter*, *Blautia*, *Ruminococcus*, *Fusicatenibacter*, *Lactobacillu* and *Anaerostipes*) and serum metabolites (NIM: 4-Methyl-2-Oxopentanoic Acid, 2-Hydroxycaproic acid, Isovaleric acid, Testosterone sulfate, Dihydrotestosterone glucuronid, and PIM: Epitestosterone, Etiocholanolone, Cotinine, Indole-3-lactic acid, Mentha-1,3,8-triene) to predict reduced BMD. As shown in **Figures 7A–C**, the individual marker panels could discriminate individuals with reduced BMD from normal individuals in both the discovery and validation sets. Importantly, the combination of the gut microbiota and serum metabolites has good diagnostic performance (**Figure 7D**).

## Discussion

Accumulated evidence has shown that dysbiosis of the gut microbiota and metabolites contributes to multiple metabolic diseases in older people, but less is known about the gut microbiota and serum metabolite signatures of postmenopausal women with reduced BMD (He et al., 2020). Over the age of 50 years, the high incidence of osteoporotic fracture has led to a significant burden for patients and health care providers. It is essential to find ideal targets for researching the risk factors and developing reduced BMD for intervention and treatment in advance. Here, for the first time, we characterized the gut microbiota profile and signatures of serum metabolites in postmenopausal women with reduced BMD. Our results indicated a clear difference in the gut bacterial composition and serum metabolite abundance between the OS group and the normal group. The specific alterations in the microbiome and derived metabolites were strongly associated with the BMD index and bone turnover markers, indicating the potential of the gut microbiome and serum metabolites as modifiable factors and therapeutic targets for preventing osteoporosis.

Alterations in the gut microbiota were associated with a reduction in bone mineral density (Das et al., 2019). The findings of our study suggested that the α diversity of the gut microbiota differed between the OS and normal groups, and the results indicated that bacterial richness and diversity were increased in the OS group. We observed that some bacteria at the genus level, such as *Bacteroides*, *Blautia*, *Fusicatenibacter*, *Ruminococcus*, and *Anaerostipes*, were enriched in the normal group and that *Agathobacter* and *Lactobacillus* were more abundant in the OS group. *Bacteroides*, which was decreased in OS patients, has been reported to ameliorate bone loss in ovariectomized mice (Yuan and Shen, 2021). Consistent with our findings, beneficial bacteria, such as *Anaerostipes* and *Blautia*, which can inhibit inflammation, were also decreased in OS patients (Benitez-Paez et al., 2020; Bui et al., 2021). A beneficial anti-inflammatory association of *Blautia* was also found in other clinical settings, including in colorectal cancer (Chen et al., 2012), cirrhosis (Qin et al., 2014), and inflammatory pouchitis following ileal pouch-anal anastomosis (Tyler et al., 2013). Furthermore, the depletion of the butyrate-producing *Firmicutes* bacterium *Fusicatenibacter* in OS patients could produce a beneficial effect on maintaining the stability of the intestinal lumen environment (Tian et al., 2023). *Ruminococcus* was regarded as a beneficial bacterium related to the prevention of osteoporosis in studies focusing on the anti-osteoporosis mechanism of active ingredients (Han et al., 2023; Xiao et al., 2023). To the best of our knowledge, *Agathobacter* has not been reported to be associated with osteoporosis. In a study identifying diagnostic biomarkers for compensatory liver cirrhosis, *Agathobacter* was elevated in patients with compensatory liver cirrhosis (Chen et al., 2023). In addition, previous studies have shown that *Lactobacillus* was more abundant in the osteoporosis group than in the control group (He et al., 2020), which was consistent with our results. Moreover, another study showed that *Lactobacillus* supplementation could reduce bone loss in older women with low bone mineral density. We also found a significant alteration in *Lactobacillus* abundance in OS individuals, but the opposite effect was observed (Nilsson et al., 2018; Li P. et al., 2022). Conflicting results may be explained by the number of specimens and populations included in these studies. As our study demonstrated, the gut microbiota has a close relationship with bone turnover markers (Chen et al., 2021), which is consistent with the increase in bone turnover markers observed in the OS group.

In this study, T-PINP, β-CTX and N-MID were increased in the OS group compared with the normal group, consistent with the findings of previous studies (He et al., 2020; Wang et al., 2023). In addition, our data demonstrated a positive association between the *Bacteroides*/*CAG-352*/*[Eubacterium]_siraeum_group* and BMD and a negative association between the *Agathobacter*/*Lactobacillus* ratio and BMD. By LEfSe analysis, we also observed that *Bacteroides*/*CAG-352*/*[Eubacterium]_siraeum_group* were increased in the normal group, and *Agathobacter*/*Lactobacillus* was increased in the OS group, which correlated well with the correlation analysis of the BMD index and gut microbiota. In the present study, we also performed a functional prediction analysis of the gut microbiota and found that the pathways involved in the biosynthesis of terpenoids and steroids, photosynthesis−antenna proteins and carotenoid biosynthesis were enhanced in the OS group. Further correlation analysis of the gut microbiota and metabolic pathways revealed that the biosynthesis of terpenoids and steroids and the metabolic function of photosynthesis-antenna proteins were positively correlated with *Agathobacter* and *Lactobacillus*, which were increased in the OS group; moreover, *Bacteroides* and *[Eubacterium]_siraeum_group*, which were increased in the normal group; and maintained a negative association with the abovementioned pathways. In addition, many studies have demonstrated that the gut microbiota regulates the immune system in a similar way to bone metabolism (D’Amelio et al., 2008; Hsu and Pacifici, 2018). A decrease in microbial dysbiosis and downregulation of inflammatory signaling pathways have been associated with increased Bacteroides abundance, and all these factors could influence the immune system, leading to the amelioration of bone loss and microstructural destruction (Yuan and Shen, 2021). These results indicated that some gut bacteria are closely associated with bone metabolism, but further studies are needed to determine the underlying mechanisms involved in bone loss.

Moreover, untargeted serum metabolism analysis revealed lower levels of epitestosterone, etiocholanolone, testosterone sulfate, indole-3-pyruvic acid and isovaleric acid and higher levels of stiripentol, 7-ketocholesterol and adipic acid in the OS group than in the normal group. In addition, the altered metabolites were mainly enriched in fatty acids and conjugates, tryptamines, indolyl carboxylic acids, amino acids and peptides. Furthermore, the enrichment of relevant metabolic pathways that differed significantly between the two groups was based on differentially abundant metabolites, which indicated the enrichment of tryptophan metabolism, the biosynthesis of unsaturated fatty acids, steroid hormone biosynthesis, fatty acid degradation and the biosynthesis of valine, leucine and isoleucine. It has previously been demonstrated that etiocholanolone can enhance osteoblast proliferation (Wu and Zhang, 2018), and the present study confirms these findings. The metabolites of dehydroepiandrosterone (DHEA), particularly estrogen and testosterone, play an important role in bone homeostasis, and a significant link has been found between DHEA and increased bone mineral density. In part, this is because DHEA increases the activity of osteoblasts and the level of insulin-like growth factor 1 (IGF-1) in the blood (Kirby et al., 2020). Interestingly, IGF-1 is also known to improve fracture healing. Isovaleric acid improved ovariectomy-induced osteoporosis by inhibiting osteoclast differentiation in another study, in agreement with our findings (Cho et al., 2021). 7-Ketocholesterol, which was the most common serum metabolite in the OS group, was recently reported to induce oxiapoptophagy and inhibit osteogenic differentiation (Ouyang et al., 2022). In terms of metabolic pathways, tryptophan and its metabolites in the regulation of bone metabolism have been well investigated in numerous studies (Michalowska et al., 2015; Al Saedi et al., 2022; Tsuji et al., 2023), and some tryptophan metabolites could become important targets for developing new pharmacological treatments for osteoporosis. Additionally, a multiomics study in a large cohort identified an amino acid metabolism-mediated association between gut microbiota and osteoporosis, suggesting that gut dysbiosis and amino acid metabolism could be a target for osteoporosis treatment (Ling et al., 2021). Recent research suggested that puerarin may promote the biosynthesis of unsaturated fatty acids and regulate phospholipid metabolism in OVX-induced osteoporosis (Li B. et al., 2022), and the results showed that biosynthesis of unsaturated fatty acids was important in undermining adipogenic differentiation and promoting osteogenic differentiation of BMSCs (bone marrow mesenchymal stem cells) in ovariectomized rats. Although the relationship between steroid hormones and osteoporosis has long been established, relevant research has indicated that probiotics can prevent sex steroid deficiency-induced bone loss (Li et al., 2016). These findings appear to be consistent with our results, and much of the relevant literature has clarified the important role of the gut microbiota in osteoporosis progression.

Among the mentioned metabolic pathways, steroid hormones are uniquely metabolized by the host, and fatty acid degradation is cometabolized by the host and gut microbiota. Importantly, host- and gut microbiota-derived metabolites jointly participate in the tryptophan metabolism pathway, and the plasma-derived indole is uniquely metabolized by the gut bacteria-derived *tnaA* gene. Moreover, the predicted abundance of the *tnaA* gene in the OS gut microbiota was significantly lower than that in the normal gut microbiota, and the *Bacteroides* genus was strongly correlated with the *tnaA* gene. By comparing the abundances of gut microbes that belong to the *Bacteroides* genus at the species level, we found that the abundances of *Bacteroides stercoris*, *Bacteroides plebeius*, and *Bacteroides coprocola* were significantly decreased in the OS group. Among them, only the abundance of *Bacteroides stercoris* was positively correlated with the abundance of the *tnaA* gene. To the best of our knowledge, the *Bacteroides stercoris* and *tnaA* genes have never been reported to be associated with osteoporosis, indicating that further studies are needed to investigate the potential action and underlying mechanisms of these genes on osteoporosis.

Correlation analysis allowed us to identify the associations between gut bacteria and serum metabolites and bone mineral density indices. We found positive associations between normal group-enriched *Bacteroides* abundance and normal group-enriched epitestosterone, etiocholanolone and testosterone sulfate. In addition, *Bacteroides* correlated positively with femoral neck and hip BMD, and a worthwhile indicator, testosterone, correlated positively with lumbar spine BMD. In this study, we also focused on the early diagnostic value of gut bacteria/serum metabolites for reduced BMD. Using the linear regression model, we confirmed that the presence of gut bacteria combined with serum metabolites could be a better indicator for predicting reduced BMD. A mathematical regression model was recently used as a practical method for the early diagnosis of postmenopausal osteoporosis after screening with multiple feature selection methods (Kwon et al., 2022). Individual marker panels of gut bacteria or serum metabolites could discriminate individuals with OS from healthy individuals in both the discovery and validation sets. Importantly, the combination of gut bacteria and serum metabolites had a greater diagnostic performance. Over the age of 50 years, the incidence of osteoporotic fracture is approximately 1/3 and 1/5 in female and male populations, respectively, during their lifetime, representing a significant burden for patients and health care providers. Therefore, it is highly important to effectively predict the occurrence of osteoporosis at the early stage (Munoz et al., 2020).

## Conclusion

Collectively, our results suggested that postmenopausal women with reduced BMD have significant changes in the gut microbiota and serum metabolites, which are significantly correlated with the BMD index and bone turnover markers. This correlation provides potential directions for exploring the mechanism of osteoporosis development and potential early diagnostic indicators for reduced BMD. This study might lead to the use of novel interventions to improve the level of bone health in postmenopausal women.

## Data availability statement

The data presented in this study is deposited in the NCBI repository, accession number PRJNA1084172.

## Ethics statement

The studies involving humans were approved by Ethics Committee of The Second People’s Hospital of Dalian. The studies were conducted in accordance with the local legislation and institutional requirements. The participants provided their written informed consent to participate in this study. Written informed consent was obtained from the individual(s) for the publication of any potentially identifiable images or data included in this article.

## Author contributions

LY: Funding acquisition, Writing – original draft. XW: Writing – original draft. TY: Investigation, Software, Writing – review & editing. ZQ: Data curation, Writing – review & editing. HL: Investigation, Software, Writing – review & editing. HN: Formal analysis, Writing – review & editing. KW: Methodology, Project administration, Writing – review & editing. DL: Resources, Validation, Writing – review & editing. FH: Conceptualization, Investigation, Supervision, Writing – review & editing. WW: Funding acquisition, Resources, Writing – review & editing.
